# Changes in eating attitudes and behaviours in a sample of female university students studying health degrees: a 12-months follow-up study.

**DOI:** 10.1186/2050-2974-3-S1-P9

**Published:** 2015-11-23

**Authors:** Tetyana Rocks, Fiona Pelly, Lisa Martin, Gary Slater

**Affiliations:** 1 University of the Sunshine Coast, Sunshine Coast, Australia

## 

It remains unclear if studying a health-related degree alters an individual's eating attitudes and diet-related behaviours. This study explored changes in eating attitudes and behaviours in 36 female students (mean age 28.6 ±10.0 years) enrolled in health degrees (Nutrition and Dietetics, n=26; Occupational Therapy, n=10) over a year of their undergraduate studies. Participants were asked to complete several self-reported questionnaires, including the Eating Attitudes Test (EAT-26), the Three-Factor Eating Questionnaire (TFEQ-R18), the Rosenberg's Self-Esteem Scale, and the Body Shape Questionnaire (BSQ-8D) that assessed their demographic, anthropometric, dietary, body satisfaction and self-esteem related characteristics in September-October 2013 and again 12 months later. Results suggest a significant decrease in eating disorder risk and body dissatisfaction, with an increase in self-esteem. However, the participants' weight, cognitive restraint, uncontrolled eating and emotional eating remained stable. The associations between the explored characteristics will be presented on a group and individual level. Possible directions for future research in this population will be outlined.

